# Agent-Based Modelling for Simulation-Based Design of Sustainable Faecal Sludge Management Systems

**DOI:** 10.3390/ijerph16071125

**Published:** 2019-03-28

**Authors:** Adrian Mallory, Martin Crapper, Rochelle H. Holm

**Affiliations:** 1 School of Water, Environment and Engineering, Cranfield University, Cranfield MK43 0AL, UK; A.Mallory@cranfield.ac.uk; 2 Department of Mechanical and Construction Engineering, Northumbria University, Ellison Place, Newcastle Upon Tyne NE1 8ST, UK; 3 Centre of Excellence in Water and Sanitation, Mzuzu University, P/Bag 201, Mzuzu 2, Malawi; Rochelle@rochelleholm.com

**Keywords:** agent-based modelling, design, faecal sludge, reuse, sub-Saharan Africa, system

## Abstract

Re-using faecal sludge (FS) to generate value has the potential to contribute towards solving the issue of long term sanitation solutions in growing urban areas across sub-Saharan Africa; however, hitherto, no design tools have been available that are capable of simulating a system involving economic factors, complex social issues and environmental circumstances. We hypothesized that Agent-Based Modelling (ABM), when deployed with appropriate rigour, can provide such a tool. Extensive field work was carried out in a Malawian city, investigating the adoption of Skyloo above-ground composting toilets by households, and the operation of the municipal FS site. 65 semi-structured interviews and 148 household interviews, together with observations, were carried out to characterize these processes, with the data acquired being used to construct two separate ABMs. The Skyloo ABM was run for various scenarios of start-up capital for business and payback of loans against the toilet cost to households. The municipal FS Site ABM was run for different patterns of dumping fee and enforcement structure. The field work demonstrated that there is potential for further expansion of FS reuse, with a market for agricultural application. The Skyloo ABM identified the significance of start-up capital for a business installing the toilet technology; the municipal FS Site ABM showed that existing fees, fines and regulatory structure were insufficient to reduce illegal dumping of FS to any useful degree, but that a monthly permit system would provide enhanced revenue to the city council compared with per-visit charging of disposal companies at the municipal FS site. Whilst each ABM ideally requires some additional data before full application, we have, for the first time, shown that ABM provides a basis for the simulation-based design of FS management systems, including complex social, economic and environmental factors.

## 1. Introduction

More than 80 per cent of wastewater from human activities is discharged into the rivers or sea without any pollution removal [1]. The Sustainable Development Goals (SDG) aim to halve this proportion and increase recycling and reuse globally [1]. Faecal sludge (FS) treatment plants in developing countries often fail following construction due to low operational capacity and lack of financing for maintenance [2]. Increasingly, the private sector has been seen as a solution to these issues particularly with models of resource recovery [3,4]. Yet, there are questions around the focus on private sector solutions for sanitation systems, particularly in its limitations in serving poorer population members [5,6].

Suitability of FS recycling to generate economic value as a profitable operation depends on the context in terms of local culture and existing infrastructure, perceptions of waste-based products and availability of competing products, whilst changes in use of technology and operation and maintenance of infrastructure are dependent on economic and social factors that need to be integrated into design and planning [7,8,9]. Currently, however, no tool is available to allow the combination of social and political factors, as well as any technical issues, to be addressed in the design of a FS management system.

Malawi, a low-income country in sub-Saharan Africa, has challenges in terms of providing safe sanitation and FS treatment in cities. 33% of the population relies on either unimproved sanitation facilities or practices open defecation [10]. In Malawi, illegal disposal and reuse by people in areas surrounding disposal sites is practiced [11]. Increasing urbanization and limited property space in Malawi makes the emptying of pit latrines, the most common form of household sanitation facility, an increasing requirement [12,13]. In response to these issues, projects have implemented Skyloo facilities that can be managed at household level, and produce compost for agricultural use. Skyloo pit latrines (ecological sanitation) are an adaptation of the Fossa Alterna, which use two dry composting pits above ground surface that can be alternated, whilst allowing the other pit to produce compost [14]. Skyloos are similar to the Fossa Alterna, but use raised pits that guard against problems of flooding and pit collapse common in areas of high groundwater.

Looking at technological change, either to decentralized Skyloo systems or in re-designing of centralized treatment plants, Agent-Based Modelling (ABM) offers a possible tool to assess the interaction of social, technical and economic factors. ABM has been explored to look at scenarios of technology and infrastructure change such as: the transition from centralized sanitation to decentralized composting toilets in East Germany [9], infrastructure management in political systems [8], and adoption of new technologies across a social network [15,16,17]. These models provide a framework for looking at adoption of technology; however, most do not present a method of data collection, building a model grounded in data, and then validating results. Panebianco and Pahl-Wostl provided the closest suitable model for decentralizing sanitation and adoption of technology [9], but did not present the modelling results and then the re-calibrating that would come after, to create a working design tool.

Based on the gaps in knowledge around the potential for reuse in urban Malawi and the lack of tools to accommodate social and cultural factors into the design, we hypothesized that ABM can provide a tool for simulation-based design of sustainable FS reuse systems, if deployed with appropriate rigour. Therefore, this research aimed to demonstrate the potential of ABM as a design tool for increasing the scale of existing forms of FS reuse.

As a first stage, field work was used to understand the forms of FS reuse already happening, and how different stakeholders (donors, local government and users) interact in the management of sanitation, and the potential of different resource-based models. The collected data was then used to construct two ABMs of aspects of the sanitation system; these models were tested for a range of different system design options and the results evaluated.

## 2. Materials and Methods

Our case study was conducted in a city in Malawi in 2016 and 2017. The exact location has been withheld from publication since some of the activities reported are illegal. In the study city, there is a central municipal FS site for disposing FS, managed by the local government. This mostly receives waste from formal settlement areas and institutions with septic tanks, carried to the site by private contractors using vacuum tankers. The municipal site uses two settling ponds in rotation to dry incoming sludge, which is then intended to be sold as compost for agriculture. However, the municipal FS site was closed for rehabilitation during the time of this study, partly to improve security to prevent sludge being stolen and applied by farmers in surrounding areas.

There have also been initiatives from several NGOs and organizations to implement composting toilets as a form of improved sanitation and decentralized re-use of FS. These were mainly in the form of projects that had implemented Skyloos using different funding mechanisms (Table 1). Skyloos are then managed by households, who use the compost produced on their own land. Our research looks at issues around reuse for both the centralized municipal treatment facility and the household management and implementation of Skyloos.

Mixed methods were used in the research. Firstly, a survey was conducted using purposive sampling with urban households who used pit latrines in three neighbourhoods, with around 50 survey participants in each neighbourhood (*n* = 148). The survey asked demographic questions and about existing sanitation services, awareness and perceptions of services, and perceptions of different reuse options.

Looking at the two forms of reuse, centralized composting and projects implementing Skyloos, semi-structured interviews were used with different participant groups. Key informant interviews (*n* = 7) were conducted using purposive sampling with local NGOs, sanitation businesses and council employees who had a role in either form of FS reuse.

To understand issues around the management and use of Skyloos as a form of household sanitation and decentralized reuse, semi-structured interviews were conducted using snowball sampling with users who had adopted Skyloos (*n* = 48). A selection of Skyloo user interviewees were chosen from all known city-wide projects, with a sample of between five and 15 interviewees from each, depending on the number of people each project served, and the ability to find interviewees by snowball sampling. Skyloo users were asked about how they had been introduced to the technology, how the toilet had been financed and how they found the use of the toilet and of the compost.

To understand issues with management of the municipal disposal sites, particularly the illegal disposal and reuse by farmers in surrounding areas, semi-structured interviews were conducted using snowball sampling with farmers who applied FS from the disposal site (*n* = 11). Farmers residing adjacent to the municipal FS site and using untreated sludge in agriculture were selected until the snowball sampling led back to the people who had already either participated or not consented to the research. Farmers who used FS from the disposal site were asked about how they used it, difficulties with access and how it compared to chemical fertilizer.

Household surveys were collected by a field research team using the Open Data Kit (ODK) software (ODK community, open source), an open source software that can use mobile phones for collection of survey responses [18]. Results were compiled in Microsoft Excel^®^ (Microsoft Office 365, Microsoft Corporation, Redmond, WA, USA).

Interviews were conducted in the preferred language of the interviewee, either local vernacular or English, and transcribed within 24 h. Photographs were also used to capture individual cases representative of resulting themes. The interview transcripts were coded thematically using Nvivo (version 11, QSR International, Melbourne, Australia), according to guidelines set out by Robson and McCartan [19].

After analysis of data from interviews, two ABMs were built using the analysed data in Netlogo software (version 5.3.1, Northwestern University, Evanston, IL, USA) [20], with the output data compiled and analysed in Microsoft Excel^®^. Agents were given properties, rules and methods of interaction with each other and with the environment, all based on the data from interviews and surveys. Such properties included connection to other agents, openness to adoption of new technology, willingness to dispose FS legally or illegally, income and perception of reuse of FS. Having built rules governing behaviour and interactions of agents, the models were then run to simulate a five-year period to assess the potential for centralized reuse of pit latrine FS, and the scaling up of Skyloos as a form of decentralized reuse.

Our research had ethical approval from the University of Edinburgh and the Malawi Government National Commission for Science and Technology (Protocol P02/17/155).

## 3. Results and Discussion—Fieldwork

This section looks at the results from interviews and surveys and households with respect to FS reuse. The results in Section 3.1. refer to themes that were identified in approaches to reuse, centralized collection and treatment of waste at the municipal site, and in the management of decentralized Skyloo facilities. Section 3.2. refers to results specifically relating to the management of the municipal treatment site, particularly with farmers illegally using sludge in their own farms. Section 3.3. refers to results specifically relating to marketing, implementation and management of Skyloos in households.

### 3.1. Constraints to Improving Sanitation Services

Table 2 shows a summary of barriers to FS management. These are further discussed below.

#### 3.1.1. Sanitation Marketing

In our study, often respondents were not aware of different sanitation services and providers in the city. Not knowing of reuse and pit emptying options were the main reasons household respondents gave for not using these services. From the survey participants in three urban areas relying on pit latrines, none had used (formal or informal) pit emptying services before, with 16/19 households who had previously had a pit latrine become full not emptying it because they were not aware of available services. 64/148 households were open to the use of FS in agriculture, and 20/84 households who were against the idea cited unawareness as the reason. This lack of awareness can also be seen from the business side, with pit emptiers rarely promoting their services:

“Am well known for what am doing and people send the messages through relatives whenever they have a pit latrine that needs to be emptied.”

(Manual Pit Emptier, Male, 42)

In terms of adopting new technologies and reuse of FS, a common theme from interviewees who used Skyloos was being informed by project (Table 1) based marketing campaigns:

“So I first heard from (Project 3). From them I heard about these toilets that are taking less land and can harvest manure.”

(Skyloo User and Loan Collector, Project 3, Female, 62)

Implementing household sanitation that can either be emptied to the composting site or used for household compost would require further education and marketing. There were no cases found of Skyloo adoption through word of mouth after projects ended.

#### 3.1.2. Transport

Transport issues act as a constraint to current approaches to FS reuse, both for transporting compost from Skyloos to areas for use and for emptying and transport of pit latrine waste to the municipal site. These issues begin at household level in informal areas, where there are often narrow earthen roads that are difficult for emptying vehicles to access:

“In terms of transport for the manure as it is heavy and my farm is in (a different neighboured than their home) so difficult to have everything ready constantly carrying with a bike.”

(Skyloo User, Male, 42, Project 5)

Transport was also a reported challenge for farmers taking sludge from the municipal FS site. All 12 interviewees were subsistence farmers, and most carried the sludge either on their head or in wheelbarrows, with two farmers using a bicycle and one owning a car. Nine farmers cited the issue of transporting sludge as a challenge:

“There are accessibility problems especially in rainy season because it’s very heavy to carry while in dry season it is easy to access…”

(Farmer using sludge, Interviewee 1)

In such urban areas, the FS reuse would require nearby (within walking distance) land where compost could be used.

#### 3.1.3. Taboo of Reuse and Handling of Faecal Sludge

The issue of sanitation marketing and awareness of different solutions is also tied into the cultural taboo around FS reuse and handling, that was cited by many interviewees:

“I’m concerned that they (tenants) would not like the idea of it and using ash and things so instead I made a (traditional pit) latrine at the back. The positioning of this one is also not good and is close to the house. Maybe could discuss with them to see how they would feel but I’m concerned they wouldn’t like it.”

(Skyloo User, Project 1, Female, 59)

The lack of discussion or spread of ideas was also shown when Skyloo users from Project 4, where the organization had promised to find a market for compost, ultimately households had to find a market themselves. Most found a method to sell their manure; however, one could not despite being in a savings group with the others:

Interviewee: “I have done it before (emptied the Skyloo) 4 times now but when they built they promised they would find a market for us but now I have to dig pits myself and bury the waste each time and I’m running out of space to dig.”

Interviewer: “Have you consulted anyone else with Skyloos?”

Interviewee: “No I haven’t done. But most of them are doing the same as me and I’ve been waiting for these people to come back.”

(Skyloo User, Project 4, Male, 60)

There was also a taboo around the reuse of FS from the municipal FS site, with people feeling ashamed to be handling FS:

“So the moment the waste comes in people come to get it and people look down on you and with shame. See we have to go without gloves or gum-boots (tall plastic boots) and we feel it too but it is a need so it has to be done.”

(Farmer using sludge, Interviewee 2)

This taboo often leaves a fractured market in terms of information, causing difficulty in spreading ideas, which provides a barrier to services scaling up across the city. This means that projects may not develop beyond the scope of those to whom they can directly market, which suggests large marketing expenditure or government backing would be required to spread sanitation technologies.

#### 3.1.4. Scope and Application of Compost

Whilst the survey respondents and interviewees knew that FS had nutrient value for their crops, there was a gap in knowledge about the extent of nutrient quality and associated health risks. This resulted in varying approaches to the use of FS from the municipal site and the compost end-product from Skyloos. Six Skyloo users had previously sold compost for varying prices, two Skyloo users threw the compost away, three gave compost to neighbours and 16 used it in nearby gardens. Eight users claimed the compost could support quite large farms, and could replace the chemical fertilizer 23–21 or urea, which cost MK11,430 (USD15.77) and MK10,670 (USD14.72) per 25 kg bag respectively:

“The benefit is there of course but I am not sure how much to apply as if I apply too much it will not germinate but if it is too little then the crops won’t grow heavy.”

(Farmer using sludge, Interviewee 2)

There was also a varying perception of the risks associated with compost from Skyloo toilets and the risk of using sludge raw from the municipal FS site:

“Also needs protection such as gloves for (the composted) manure as usually people use their bare hands and even if it looks as sand people are still thinking that it is waste.”

(Skyloo User, Project 2, Female, 30)

Interviewer: “Do you feel there are there any health risks?”

Interviewee: “No I haven’t noticed. There is a pile of that so when collected it’s from a pile so it’s disgusting and produces a heat.”

(Farmer using sludge, Interviewee 5)

The perception of risk from Skyloo compost is something that would need further investigation, as there are conflicting views in the literature about its safety. The current recommendation of six months composting time is seen by [21] as unlikely to be sufficient for safety.

The lack of clear value can also be seen in the variation of the price for different sludge-based products in Malawi. Of the six respondents who reported selling manure from Skyloo toilets, 50 kg bags were reportedly sold for up to MK6000 (USD8.28), whilst in other areas some only recorded a price of MK500 (USD0.69) per bag. From city council interviewees, it was found that composted sludge from the disposal site had been priced at MK300 (USD0.41) per 50 kg bag. Due to issues of management, discussed later, the council were not able to actually produce or sell compost at the time of this study, hence, it is not clear whether there was demand at this price. To realistically identify the potential of reuse, a better understanding of the nutrient content and market value would be needed.

### 3.2. Illegality of Faecal Sludge Reuse

#### 3.2.1. Reuse of Pond Material by Farmers

There were two interviewees involved in reuse of sludge from the municipal site who did not consent to participating in the research due to the illegal nature of the activity and previous conflict with the local government. One respondent, who according to other participants had been arrested and fined MK15,000 (USD20.69) for stealing sludge from the pond, consented to participate, but said that he had only used the sludge once, but had stopped using it, despite being up to date about rehabilitation of the ponds:

Interviewer: “Would you envisage using in the future?”

Interviewee: “To me I would want to but the rehabilitation means it will be difficult to access from the heavy guarding.”

(Farmer using sludge, Interviewee 12)

As well as people who chose not to participate in the study, there was generally a suspicion of interviewers that made it difficult to understand what the current situation was. In an area where the research found three participants, we were later told by another that the entire group of around 10 people was still using sludge illegally. No common story arose about when they had been able to take sludge and when it had been stopped:

“The time when there were guards there were issues as I think when they saw us coming they thought they could make some money so after if we gave a little money we could access but if we didn’t they would say the city council doesn’t allow and that it’s a disposal site for public health. To some who didn’t understand, they stopped using it when the guard stopped them but I understood the benefit so I continued to use. Even in the future if I don’t apply my land will still be strong.”

(Farmer using sludge, Interviewee 8)

“I think there are more than 50 but don’t want to say. People wouldn’t want to be known in the open otherwise they will be afraid.”

(Farmer using sludge, Interviewee 8)

What became clear from interviewees was a sense of distrust and fear that had grown from the arrests and fines issued. It seems the local government had historically at least taken some steps to stop illegal use. Some interviewees had moved to the area more recently, and had not experienced this:

Interviewee: “So I came late when moving to this area so I applied when I was planting so I would dig holes in the area and apply it there. I have only just moved here in January.”

Interviewer: “Does the FS cost anything to get?”

Interviewee: “We’re just getting it free currently.”

(Farmer using sludge, Interviewee 1)

An issue mentioned by three interviewees was that of the competitiveness of access to the sludge, which meant that people increasingly rushed to take the sludge:

“So the first challenge is the distance. Secondly because we collect individually there is no communication to share ideas or be able to co-ordinate instead of the way it happens where people simply take”

(Farmer using sludge, Interviewee 3)

While the scale of illegal reuse is not clear from the interviews, it is clear it is happening. However, often, respondents did not want to refer others to the research, meaning the snowball sampling was limited. The indication that bribes were being paid is strong evidence that reuse had value to somebody. It seemed there were around 15–20 farmers practicing illegal reuse in the area, and potentially more in surrounding areas.

#### 3.2.2. Public Health Risk

The practice of taking raw sludge from the ponds has clear health risks [2], with children also being responsible for tasks involving FS in some families, although no interviewee cited health problems when asked about pathogens or health risks. This is different from the situation in Ghana, where 24% of farmers using raw FS reported health problems associated with FS application [22]. Beyond the farmers using sludge, there could be a risk of food contamination [23], though only two farmers sold any produce, with the rest using crops for their own household consumption:

“To me it’s difficult as mainly I grow vegetables that I sell at hospitals and different areas so my supply contract is with council. So if I’m using the manure it compromises the business.”

(Farmer using sludge, Interviewee 12)

As it is currently managed, the reuse of raw sludge poses a risk to the farmers involved, both from the faecal contamination, but also from other wastes that can be mixed in:

“The main issues were with the risks of handling as there could be condoms, syringes and other wastes in there.”

(Farmer using sludge, Interviewee 10)

The use of plastic bags as basic gloves was the highest level of personnel protective equipment cited, and the sorting of glass and syringes suggests risk to the health of the people currently practicing reuse. This highlights the need for improved protection if reuse is to be safe.

#### 3.2.3. Issues of Management of Disposal Site

There is a series of issues with the management of the ponds. The first is the balance between cost recovery for sustainability and the encouraging of good practice. The existing system discourages good practice, as the fines for illegal disposal are lower than the fees for legal disposal as discussed by [24].

### 3.3. Constraints to Adoption and Continued Use of Skyloos

**Figure 1:**
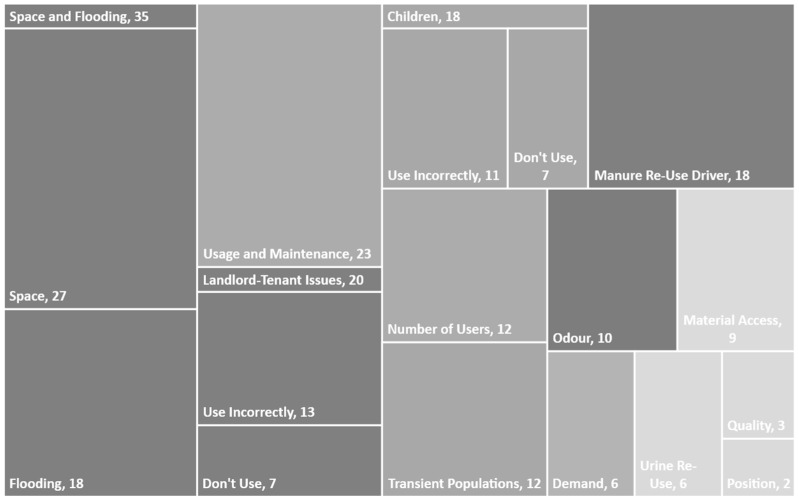
Themes from Skyloo Interviewees.

Beyond the general constraints to the whole sanitation sector that inhibit the development of reuse in agriculture, there are many issues specific to the use of the Skyloo latrine design. Figure 1 shows the themes mentioned by Skyloo users in our study, with the area of the boxes corresponding to the number of interviewees who mentioned the issue. There were many issues of poor maintenance found with farmers (23 mentions), which often related to either a lack of defined responsibilities between landlords and tenants (20), too many users (12), a high turnover of tenants leading to people who were not used to the technology (12) or children struggling to adjust to the technology (18). Skyloos were adopted to deal with the issues of space and flooding in pit latrines (35), and sometimes due to the high construction quality (2). It was noted that some households were specifically attracted to the ability to produce compost (18), though complaints existed around finding a market demand for compost (6), how to manage diverted urine in Skyloos that used urine diversion (6), bad smells (10) and getting materials to maintain the Skyloo (9). Other issues noted related to the positioning of the latrine causing issues in management (2).

#### 3.3.1. Financial Constraints

The expense of constructing Skyloos is a challenge for most people currently. This has two components: the inflation of the price being attributed to a development project, and the lack of access to finance for households. Only one user out of 47 interviewees had bought the Skyloo upfront. The provision of project loans allowed people to pay over a longer period, making the technology affordable in the context of a temporary and controlled development project. This current price has also risen from MK35,000 (USD48.28) in 2007 through to MK200,000 (USD275.91), with some respondents citing even higher prices (though this could not be verified by researchers). Without access to financial services such as loans, many customers will not be able to afford the price unless there is a donor subsidy, since the minimum monthly wage is MK20,000 (USD27.59).

The loans from Project 3 were charged at 1% interest per month, with a payback period of two years. If this system were to be reinstated with the new price of MK200,000 (USD275.91) being covered, then a monthly payback of MK9320 (USD12.86) would be needed, 22% of the average income of survey participants. Of the Skyloo users and survey participants who could give data for cost and lifespan of latrines, only 5/38 were effectively paying more than MK9320 (USD12.86) per month for a latrine.

From snowball sampling and key informant discussions, it was not possible to find any Skyloo users who had adopted the technology after the development projects in the study area after it had stopped, showing both the need for financial products and the importance of marketing from the organizations in spreading the technology. Additionally, it seems that the loan system and set-up of demonstrations did not work to trigger ongoing spread of the technology; instead, interest ended with projects.

#### 3.3.2. Space and Flooding Issues

For Skyloo users, the issue of space for digging new pit latrines and the flooding and collapse of unlined pit latrines was a bigger driver for adoption than the reuse of manure. 18 interviewees mentioned the benefit of manure as a driver for adopting Skyloo technology, whilst 35 mentioned either flooding or space or both issues, with 27 citing the permanence of the technology and not having to dig new latrines, and 18 mentioning the above ground system preventing flooding:

“There is a big difference in terms of the land use. With pit latrines I have to build another and another depending on the water level. This is a huge problem here as the water is very high. I have another plot nearby with a pit latrine with issue.”

(Skyloo User, Project 3, Male, 65)

There are two main implications of this driver for the Skyloo latrine design being more important than manure reuse. Firstly, it means that there may not be a huge scope for the technology in other areas of the city, or other regions in Malawi, that are less densely populated or less exposed to flooding. Secondly, the seemingly secondary priority of manure reuse implies that people may not be particularly concerned about the safe management of the composting process and simply want something hygienic that does not collapse. This was mentioned by two users who did not reuse compost:

“So from 2008 we harvest once a year but we just throw it and pile it at the back.”

(Skyloo User, Project 5, Female, 23)

This issue could perhaps be improved if there was a greater understanding of the market potential and demand for the compost, so that people would be encouraged to sell compost rather than simply discarding it.

#### 3.3.3. Social Capital

Though there were no local projects implementing household Skyloos at the time of the study, the legacy of the identified historical projects may make it difficult for projects in the future. This is particularly prominent with Project 4, which was stopped before many toilets were completed, with people reporting that they could no longer find the organization that ran the project. This is despite the study researchers identifying it as still being active on sanitation projects in the city:

“The (project organization) came to me with the (Skyloo) idea. They came here through the council as in this area toilets are often waterlogged. They came with the council and (local) university so I was expecting you would come to see me some time. The council owns this land. So we were told we should bring sand and bricks and then get a loan of MK25,000 (USD34.49). We were told we should be in groups and have an account for managing. Then they took the money and vanished but I’d already finished the loan and the toilet.”

(Skyloo User, Project 4, Male, 71)

This presented issues for conducting the research, as the researchers were often perceived to be part of the project that had left with their money, particularly at the start, as we had been referred by a member of the project organization. One interviewee asked why it had taken so long to come and see her, as the project knew her, though the snowball sampling had only found out about this project the same day as the interview. This was often an issue with many interviews: our research was incorrectly associated with other previous projects that had left a negative reputation for other partners trying to implement Skyloos, or similar reuse models, in such areas.

## 4. Results and Discussion—Agent-Based Modelling

Having identified two forms of reuse being practised, two separate ABMs were developed based on the data collected. The first one simulated the business potential for the installation of Skyloos, taking into account various socio-economic factors affecting whether householders adopt the technology. The second one simulated possible operational models for the refurbished municipal FS site in the city. In each case, the models were intended to allow the effect of differing values of various parameters to be simulated, to achieve outcomes that were assessable against a range of criteria such as economic and social sustainability and environmental benefit. This would allow successful FS management systems to be designed. No previous implementation of ABM in the context of sanitation has pursued the whole cycle from the collection of relevant data on which to base the model to the production of results relevant to system design.

### 4.1. Skyloo ABM

#### 4.1.1. Model Structure

**Figure 2:**
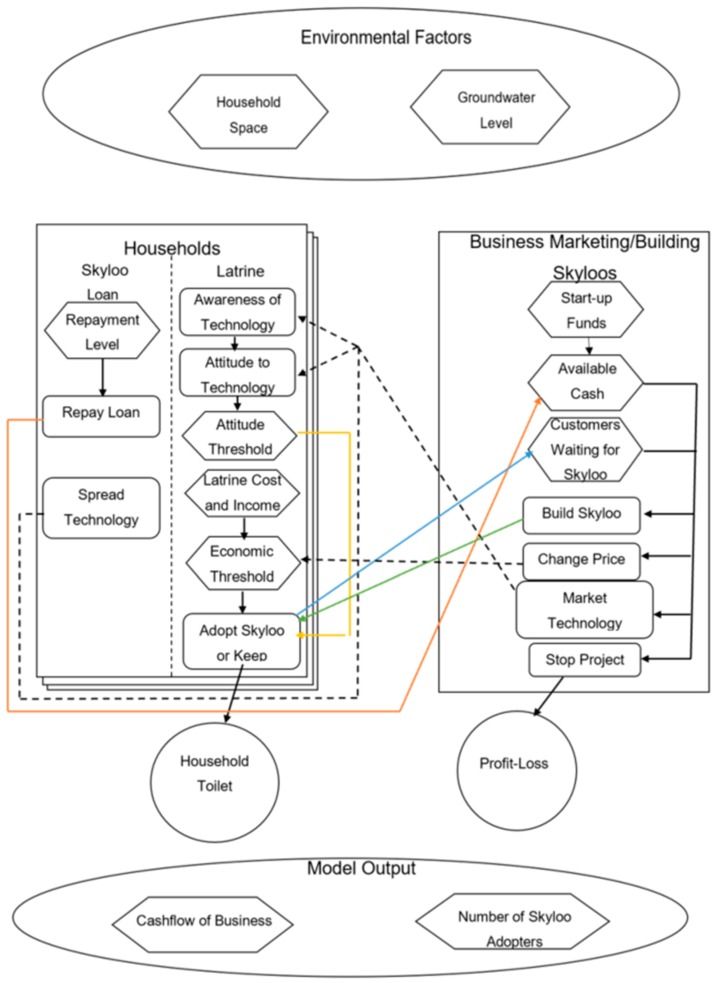
Model structure for Skyloo adoption.

The Skyloo ABM model structure is shown in Figure 2; this is broadly similar to the approach of Panebianco and Pahl-Wostl [2]. The agents are the householders who decide whether or not to adopt Skyloos, and the business which markets and builds the technology. Households that decide to have a Skyloo installed take a loan for the cost of construction, and repay it at a fixed rate.

The ABM was set up based on data captured in the field work, with a number of assumptions and limitations, bearing in mind the processing time available. Details are set out in Table 3. Households are linked in a social network along a ‘small-world’ network structure [25]. This initially links all households to their four nearest neighbours, and then rewires each link with a probability of 0.2, creating clusters of interlinked neighbours, whilst also having a small distance of links between any two sets of neighbours. This models the social environment as a small-world, where any two nodes are linked by a low number of neighbours, as would be expected in the local communities being simulated, whilst retaining the local clustering of groups that were observed in savings groups with Project 4. The code used for this part of the model was adapted from Wilensky [26]. GIS data was used to get household land-size information and distance to rivers (locally known as ‘dambo)’ and flood plain areas that were used as a proxy for flood risk.

Households either adopted the technology based on marketing from the business or hearing from linked neighbours, subject to an economic threshold defining their ability to afford to adopt Skyloos and an openness threshold defining their openness to new technology. If these thresholds were passed, then households become adopters, willing to install a Skyloo. If there were households willing to adopt Skyloos, they were added to a list of households for which the business could build. If the business had sufficient capital to build a Skyloo for a household, they would build the facility and issue a loan to the customer, with repayments beginning the next month. In some cases, people were on waiting lists for Skyloos to be built, and have not had them built due to the project financial situation, as was in the case of Project 3, where one loan collector had a waiting list of 11 households who were willing to buy a Skyloo. In the model, if households had to wait more than 30 days for a Skyloo to be built, they ‘un-adopted’ the technology.

#### 4.1.2. Model Runs

The Skyloo ABM was run for different financial scenarios. From the field work, MK9320 (USD12.86) per month was identified as the monthly repayment required for current loans; thus, in the first scenario, this was modelled as the cost of a Skyloo. In the second scenario, a charge of MK7000 (USD9.66) was modelled to see how the lower charge affected the rate of payback and success of the business.

The other variation was in the start-up capital available to the business agent: this was tested for two different values, MK10,000,000 (USD13,750) and MK2,000,000 (USD2750).

For all combinations, models were run for 1000 repeats. This is because of the various probability functions involved in setting up the small-world neighbour networks, which can have a large effect on the end results of modelling; for this reason, a large number of runs were needed to ensure the results are valid. The model was run for 2000 steps, with each step equal to a day, partly because this is within the scope of how long any Skyloo project has previously run. The use of 2000 steps is also to improve the speed of both the modelling and analysis and processing of data.

#### 4.1.3. Skyloo ABM Results

**Figure 3:**
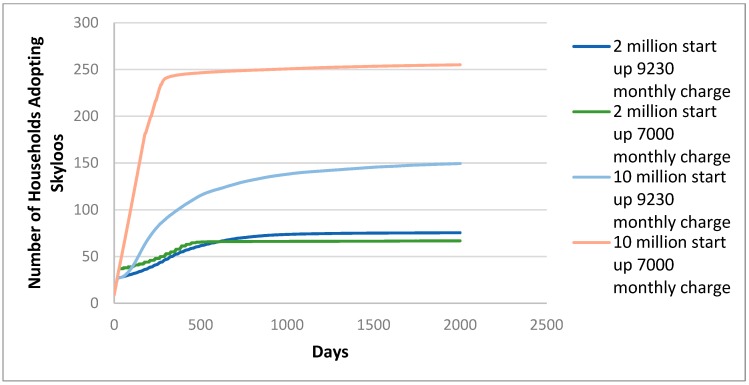
Results of ABM showing average adoption of Skyloos over 1,000 model runs based on start-up finance and monthly repayment charge in Malawi Kwacha.

Figure 3 shows the number of households adopting Skyloos for the different financial scenarios. Unsurprisingly, the greater start-up finance and cheaper payback situation gave rise to an earlier and greater adoption of the technology. It is important to note that these results say nothing directly concerning either FS reuse or environmental quality, though adoption of Skyloos might reasonably be regarded as a proxy for these, if managed correctly. Most adoptions occur earlier in the cycle; however, for both high start-up finance scenarios, there is evidence that adoptions are continuing to grow slowly even at the end of the model run, five and a half years after the inception of the business. This continuing growth is less obvious for the lower start-up capital scenarios.

**Figure 4:**
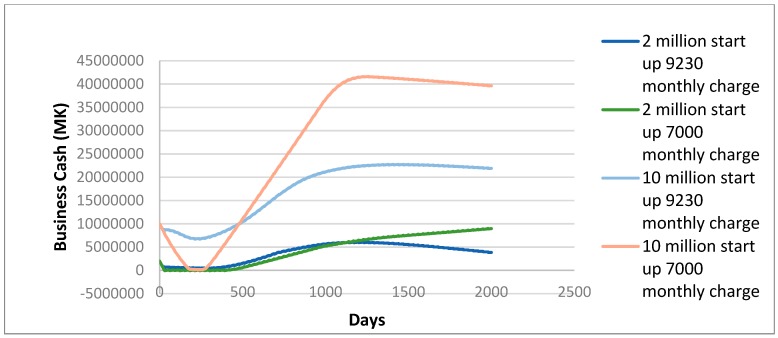
Cash flow of Business Approaches to Building Skyloos in Malawi Kwacha.

Figure 4 shows the business cash flow of Skyloo implementation in different finance and price scenarios. The level of finance is vital to cash flow and adoption levels, as in the MK10,000,000 start-up fund a business can satisfy more demand before customers ‘unadopt’ technology. The higher start-up finance situations experience an early ‘dip’ but end up with robust cash reserves to the business, although this is declining at the end of the model run and may be unsustainable in the very long term. The very lowest finance option with the lower loan repayment scheme is the only situation where the business cash is still growing at the end of the 2000 day period.

It is perhaps obvious that in a given area, the adoption of Skyloos would eventually saturate; thus, a business in a fixed location would have a limited life before needing to focus its efforts in a new area. However, different system design parameters may extend or reduce this limited life. It is also worth noting that the maximum adoption of 250 households in an area of 3000 households is still relatively low, thus, different forms of household sanitation will still likely remain part of the solution for FS reuse in Malawi. The profitability of MK10,000,000–20,000,000 (USD13,750–27,500) in the high start-up fund scenarios suggests there is a potential for an investor to make a profit from providing Skyloos with the correct marketing, though the large rate of inflation of the study area may influence both the effective profitability and may also require changing prices over a five year period.

The main shortcomings of the Skyloo ABM model are that it does not model the FS reuse stage and environmental quality, and that its initial data is based on field data derived primarily from adopters of Skyloos, with no equivalent study of non-adopters. This would be challenging to achieve in the field, but not impossible. The scope for data validation is also limited, as the Skyloo projects are no longer operational; thus, obtaining current data to validate the model is difficult, though the modelled adoption rate is comparable to that observed from other projects. However, the results demonstrate the importance of access to finance for businesses and affordability for households, and they clearly demonstrate potential of ABM to examine the diffusion of FS management technology in a business context.

### 4.2. Municipal FS Site ABM

#### 4.2.1. Model Structure

The second ABM model was designed to investigate various possible operational scenarios of the refurbished site when it comes back into use. The relevant agents are private companies who can choose to dump FS at the site or on land elsewhere, the site guard who collects fees for dumping and prevents illegal removal of FS and farmers who either legally purchase or illegally remove FS for application on their land. As with the Skyloo ABM, environmental quality is not modelled explicitly; however, the proper disposal of FS at the municipal FS site and its ultimate sale as compost may be taken as a reasonable proxy for this.

Based on the interview responses from stakeholders and farmers relating to the previous failures of the management of the disposal site, an ABM of the interactions around the site was built. In an ideal situation this model would then be validated against observation after rehabilitation of the site; however, at the time of the study, this was not possible as the refurbishment remained on-going.

**Figure 5:**
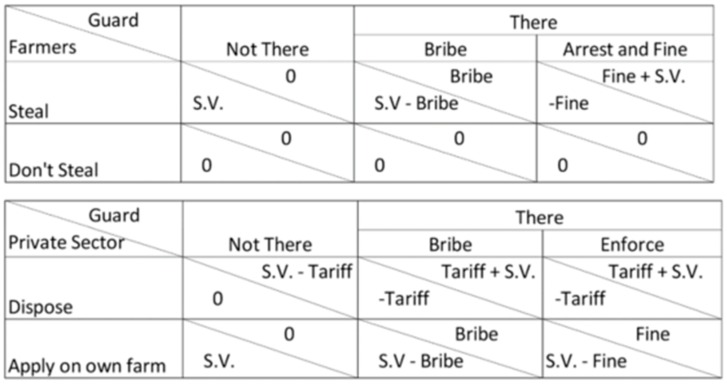
Cash flow of Business Approaches to Building Skyloos in Malawi Kwacha.

The interactions of the various stakeholders were modelled using a Game Theory approach to examine how the agents adjust their behaviour based on the relative pay-offs of different approaches [27,28]. In our model, for example, private companies have an initial probability of either illegally applying sludge on their own land or disposing at the municipal FS site, while the site guard has the choice of accepting bribes or enforcing the law. Based on the effective pay-off of each interaction, agents then adjust the probability of their behaviour to increase their chances of increasing their payoff in future interactions. There are two components to the pay-offs of different behaviours for each agent: the intrinsic value of the FS in their possession, and the loss/gain of money from paying dumping fees, bribes or fines. The pay-offs and risks of different interactions are shown in Figure 5.

A simple algorithm is adopted for adjusting the probability of behaviour, shown as pseudocode in Table 4. The multiplier is low to model a relatively slow change in behaviour.

#### 4.2.2. Modelling Approach

A range of operational scenarios were considered for the unrefurbished municipal FS site and the refurbished site. As discussed, the site was not in operation at the time of the field work; the difference between the former and proposed new states were the provision of a shelter at the entrance to encourage the guard to remain on site to collect dumping fees and sell sludge, and a fence to restrict the stealing of FS by farmers and increase the probability of their being caught and fined.

Scenarios tested including variations to dumping fees, the institution of a monthly permit system for dumping rather than a per-visit fee, the presence or absence of the guard in the unrefurbished site and bribery interactions between disposal companies, farmers and the guard. Also tested was the regulatory framework in terms of different levels of a fine for illegal dumping or stealing of sludge by farmers. Details of the values and ranges tested are shown in Table 5. Each model was run for 250 repeats over 2000 ticks, with each tick representing a day. 250 repeats were chosen as a balance of reliability of results and total processing time for all the runs.

#### 4.2.3. Municipal Site ABM Results

The most striking result of the municipal FS Site ABM is that the volume of sludge illegally disposed is essentially unaffected by any of the scenarios tested, remaining at around 6000 m^3^. This is because the relative values of all disposal tariffs and fines tested are such that the biggest payoff to the private sector companies is to dump FS illegally, whether or not they are caught and fined. Instituting a monthly disposal permit for the rehabilitated site with the guard always present increased the volume of FS safely disposed to its maximum modelled value of just over 38 m^3^ in the rehabilitated site—a tiny proportion of the illegally disposed volume. Lower values of monthly tariff and various values of per-visit dumping fee reduced this value even further. The main effect of the rehabilitation was to cut the volume of FS stolen to zero due to the presence of the fence and the guard’s 100% presence on site. The only factor that makes a difference to the guard is the post-rehabilitation situation where he is taken to be always present; this increases his income from bribes.

Since the volume of FS safely disposed relates to environmental quality, we can say that none of the scenarios tested have addressed this to any useful degree. Further increasing the fines and reducing dumping fees might have achieved improvements, and could have been modelled; however, values outside the ranges given in Table 5 above were considered unrealistic based on interviews, thus, this was not pursued. Therefore, in summary, the ABM has established that an approach other than fees and fines relating to the municipal FS site is required to gain significant improvements in the environment.

**Figure 6:**
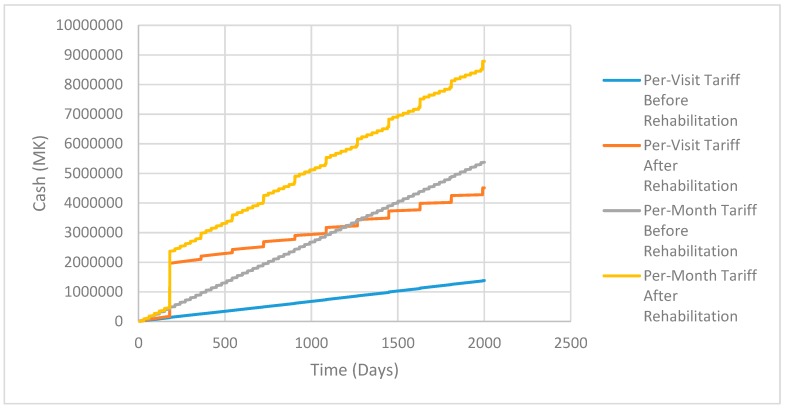
Cash flow of municipal FS Site Management.

Figure 6 shows the cash flow of different treatment approaches for the city council. A monthly permit approach is more effective at collecting revenue for the municipality, which could theoretically be used for increased enforcement or investment in infrastructure. There are increased revenues from selling compost every six months, the initial increase in cash after the first six months being notable on the figure. Compost sales income is enhanced after rehabilitation, where it is assumed the guard is present all the time, preventing theft of FS. Thus, the ABM has shown that the rehabilitated site with a monthly permit system for disposal companies provides the best option of those tested in terms of municipal revenue.

Of the model parameters that were not based on data from our field work, the FS value and guard behaviour had the greatest effect on the output when varied. This suggests these are the two major data gaps to fill to calibrate any future design and operation plan for the municipal FS site. The model could also be validated with interviews and observation after the rehabilitation of the site. Given the illegal nature of aspects of the guard behaviour, improving this data is likely to present both practical and ethical challenges; however, it is noteworthy that the ABM can consider such illegal activity as a design parameter, which would be difficult in a more traditional design approach.

## 5. Conclusions

Extensive field work has been carried out in a city in Malawi, investigating attitudes to the adoption of Skyloo composting toilets and the operation of a municipal faecal sludge treatment site. Our field work has established that there is potential for FS reuse in a city in Malawi to be expanded. Many people surveyed were found to be open to the reuse of FS in agriculture; from the farmers and pit emptiers who also use FS in agriculture, there was a clear understanding that there is a soil fertility benefit. In appropriate conditions, the implementation of Skyloos was seen to have continued use beyond the end of the implementing projects, as has been found for other toilet technology in Malawi [29]. Access to finance is clearly very significant, whilst external factors such as space availability and flood risk are also in the mix. For a system involving centralized collection and treatment of FS to create saleable compost, the relationship between private sector disposal companies, farmers who will buy (or steal) FS compost and the regulatory and fee structure and its enforcement mechanism are crucial. A successful solution will also need to maximize the volume of FS that is properly managed, rather than illegally dumped.

The field work has provided data for the construction of two Agent-Based Models (ABM), one for Skyloo marketing and installation and one for the operation of a municipal FS site. These models considered a range of technical and economic factors as well as social issues including illegal activity.

The Skyloo ABM identified the significance of start-up capital for a business installing the toilet technology; the municipal FS Site ABM showed that existing fees, fines and regulatory structure were insufficient to reduce illegal dumping of FS to any useful degree, but that a monthly permit system would provide enhanced revenue to the city council compared with per-visit charging of disposal companies at the municipal FS site.

Each ABM requires additional data before full application, which in some cases will be challenging. The Skyloo ABM requires greater information about households who have refused to adopt the technology, whilst the municipal FS Site ABM requires a better understanding of illegal dumping and bribing of the site guard.

This research has, for the first time, demonstrated that ABM models provide a method for the simulation-based design of FS management systems, including social, economic and environmental factors, based on real data and providing results to enable the evaluation of alternative system configurations.

## Figures and Tables

**Table 1:** Observed Approaches to Implementing Skyloos.

Project	Financing Approach	Material Contributions by User	Targeted User	Year of Project
1	100% subsidized by donor	No	Urban families of orphaned children through faith-based organization	2014–2015
2	Loan for house and Skyloo combined	No	Urban poor	2010
3	Loans to households from donor fund for urban development	No	Urban residents	2010–2016
4	Loans to households with donor collateral	Bricks and sand with optional further contribution	Urban residents	2012
5	Loan for house and Skyloo combined	Mud bricks	Urban poor without housing	2007–2010

**Table 2:** Barriers to Faecal Sludge Management. FS: faecal sludge.

Stage of FS Chain	Physical/Environmental Barriers	Financial Barriers	Political Barriers	Social Barriers
Household Sanitation	Latrines often flood or collapse	People struggle to afford improved sanitation without finance source		Lack of awareness of products Landlord-tenant relationship often leads to tenants using pit latrines Skyloos not suited to poorest and physically disabled Abandoned projects reduce trust in community
Collection	Poor access to some informal areas Poor road condition approaching municipal FS site	Cost of emptying is often prohibitive for households		Lack of awareness of services
Transport		Private sector dump elsewhere to avoid fees	Council unable to enforce safe disposal	People often shamed for handling FS
Treatment	No fence to prevent access	Disposal tariffs deter businesses from safe disposal Tariff not based on volume can have effect for smaller customers at household level	Unable to prevent stealing of sludge and public walking through site No direct management of funds for maintenance	Guard does not have facility or authority to collect fees Difficulty enforcing rotation of ponds
Reuse	Transport of manure is heavy and expensive	Unclear financial value of product	Reuse unsuited to poorest and disabled members of society	Disconnected market for selling compost Limited awareness in how to apply in agriculture

**Table 3:** Skyloo Agent-Based Modelling (ABM) Details of Model Setup.

Model Step	Set Up Details	Justification
Number of Households	3026	Balance of sample size with processing time
Owner/Tenant Ratio	50/50	Data from city council in case study city
Household Size (Mean/Standard Deviation (SD))	6/2	Field work found mean of 5.44 and SD of 2.3
Probability of Already Having a Skyloo	0.3%	Based on field work covering 100 Skyloos
Household Monthly Income (Mean/SD)	MK44,000/MK44,000 (USD61/USD61 approximately)	Field work identified mean as MK43,500 and SD as MK43,700
Probability of openness to reuse of FS	64/148	Based on answers in field work
Skyloo Building Cost and Capacity	Cost MK200,000 (USD276) including cost of mason Business can build 1 per day	Based on answers in field work for Project 4
Business Operating Cost	MK85,000 (USD117.26)	Based on wages for three to four people
Probability of a household gaining knowledge and becoming open to Skyloos and FS reuse	25% from marketing by business 3% from neighbours 0.8 multiplier if tenant Multipliers on a sliding scale for proximity to flood risk areas and for small houses	It was not possible to derive a %age success value from marketing as it was not clear how many people had initially been marketed to reach 50 adopters. It was evident that marketing was much more successful than neighbour communication and that tenants were less likely to adopt. Houses at risk of flooding or small houses are more likely to adopt due to space or water table issues affecting standard latrines.
Business initial contacts	300 households	Based on field work data including photos shown by past projects and interviews with marketing staff

**Table 4:** Pseudocode of Probability Adjusting Algorithm in Game Theory Approach for municipal FS site ABM.

IF actual payoff < potential payoff THEN set probability of behaviour = previous probability − 0.000001 * (potential payoff − actual payoff) ELSE IF actual payoff > potential payoff THEN set probability of behaviour = previous probability + 0.000001 * (potential payoff − payoff) END IF

**Table 5:** Model Parameters and Ranges for ABM of municipal FS Site.

Item	Initial Value with Reason	Range Tested
Dumping Charges	Observed Per Visit Charge of MK9,000 (USD12.5)	MK6000–MK15,000 Also tested were monthly permit charges of MK40,000–MK100,000
Sludge Value (per 50 kg bag)	Assumed at MK5000	MK500–MK10,000
Bribe for Guard	Assumed at MK3000	Multipliers of 1 to 8 to explore value of money paid direct to Guard compared with collecting money for authorities
Fine to Companies for Illegal Dumping	Noted as MK2000	MK2000–MK15,000
Fine to Farmers for Stealing Sludge from municipal FS Site	Based on Interviews, with some allowance for inflation, as MK20,000	N/A
Probability of Guard Presence	Not estimated as site was not in operation during field work	Assumed Unrefurbished site 65% Refurbished site 100% No shelter was available at the unrefurbished site
Time to produce saleable compost from FS	Assumed as six months based on time from sludge entering municipal FS Site to production of compost	N/A
